# Gut and Joint Microbiome and Dysbiosis: A New Perspective on the Pathogenesis and Treatment of Osteoarthritis

**DOI:** 10.3390/pathogens15010062

**Published:** 2026-01-07

**Authors:** Paulina Plewa, Patryk Graczyk, Karolina Figiel, Aleksandra Dach, Andrzej Pawlik

**Affiliations:** 1 Department of Physiology, Pomeranian Medical University, 70-111 Szczecin, Poland; paulina.plewa@op.pl (P.P.); kfigiel344@gmail.com (K.F.); dach.aleksandra@icloud.com (A.D.); 2 Faculty of Medicine, Poznan University of Medical Sciences, Fredry 10, 61-701 Poznań, Poland; patryk.graczyk@usk.poznan.pl

**Keywords:** osteoarthritis, microbiome, bacteria, gut–joint axis

## Abstract

Osteoarthritis (OA) is one of the most common and burdensome musculoskeletal disorders and a major cause of pain, disability, and reduced quality of life worldwide. In recent years, increasing attention has been paid to extra-articular factors influencing its development and progression, opening new avenues of research into pathophysiological mechanisms and potential therapies. One of the most promising areas concerns the role of the gut–joint axis and related alterations in the gut microbiome. Numerous studies indicate that an imbalance of gut bacteria, increased intestinal permeability, and low-grade inflammation may contribute to the progression of degenerative joint processes. The mechanisms through which the microbiota influences the immune system and host metabolism are becoming increasingly well understood, including pathways involving short-chain fatty acids, tryptophan metabolites, and bile acids. Despite growing evidence linking dysbiosis to the pathogenesis of OA, effective therapeutic strategies based on microbiome modulation remain under active investigation. Among the most frequently studied approaches are probiotics, dietary interventions, and more advanced strategies such as gut microbiota transplantation and targeted modulation of microbial metabolites. However, before these methods can become part of routine treatment, extensive clinical trials and a clearer understanding of causal relationships between the microbiome and joint degeneration are required. This article summarises the current state of knowledge regarding the role of the gut microbiome in osteoarthritis, outlines key research findings, and highlights current and potential therapeutic directions.

## 1. Introduction

Osteoarthritis (OA) is one of the most commonly diagnosed joint diseases worldwide [1]. It is classified as a chronic condition characterised by degeneration and atrophy of joint cartilage, mainly driven by degradation of the extracellular matrix [2]. Moreover, OA concerns inflammation of the synovium and affects the subchondral bone. OA can develop in virtually any joint, but it primarily affects the diarthrodial joints, including the knees, hands, and hips [3,4]. As the disease progresses, joint failure occurs, leading to persistent pain. Moreover, OA contributes to disability, occupational incapacity, and a marked deterioration in overall mental health [5]. According to available data, OA may affect more than 7% of the population, placing a substantial burden on healthcare systems [6]. There is little doubt that OA pathogenesis is largely shaped by the interaction of three factors: genetic predisposition, ageing, and environmental influences [7]. Considering that knee OA, genetic factors account for less than 50% of risk, researchers have increasingly turned their attention to non-genetic contributors, such as obesity and diet [5,8]. Consequently, growing interest has been directed towards understanding the role of the microbiome in OA pathogenesis. The microbiome is defined as the collection of all microbial ecosystems inhabiting various areas of the human body [9], encompassing the total microbial genetic material together with the by-products of microbial metabolism [4]. This review summarises recent and influential publications exploring the interplay between the immune system and gut microorganisms, and the metabolites through which they may influence OA.

## 2. Microbiome: Biological Basis

The digestive tract is one of the most studied regions of the human body in terms of the microorganisms that inhabit it [10]. These microorganisms encompass more than 3 million genes and nearly 5000 bacterial species [4]. The vast majority of intestinal bacteria reside in the colon [11]. They are generally classified into three main phyla: Bacteroidetes, Firmicutes, and Actinobacteria [12]. Although the gut microbiome is an extremely complex ecosystem, its composition is known to be unique to each individual, functioning almost like a biological ‘fingerprint’ [13]. The general pattern of colonisation is established in early childhood and depends on factors such as mode of delivery (natural birth vs. caesarean section) and method of feeding (breastfeeding vs. formula). The composition of the microbiome changes rapidly in the following years, stabilising in adulthood, and undergoing further shifts in older age [14]. From a population perspective, the gut microbiome remained relatively stable until the beginning of the industrial era [15]. The introduction of antibiotic therapy, the increased frequency of caesarean sections, and the widespread availability of highly processed foods have contributed to reduced consumption of complex carbohydrates, decreased species diversity, and a diminished ability of the microbiome to ferment plant polysaccharides [16].

Joints, by contrast, are considered a sterile environment in which no microorganisms are present under normal conditions. However, a gut–joint axis has been proposed, whereby neoangiogenesis permits bacteria and their metabolic products to translocate from the bloodstream into cartilage and subchondral bone [4].

## 3. Gut–Joint Axis: Mechanisms Linking the Microbiome with OA

### 3.1. Gut–Joint Axis

**Figure 1:**
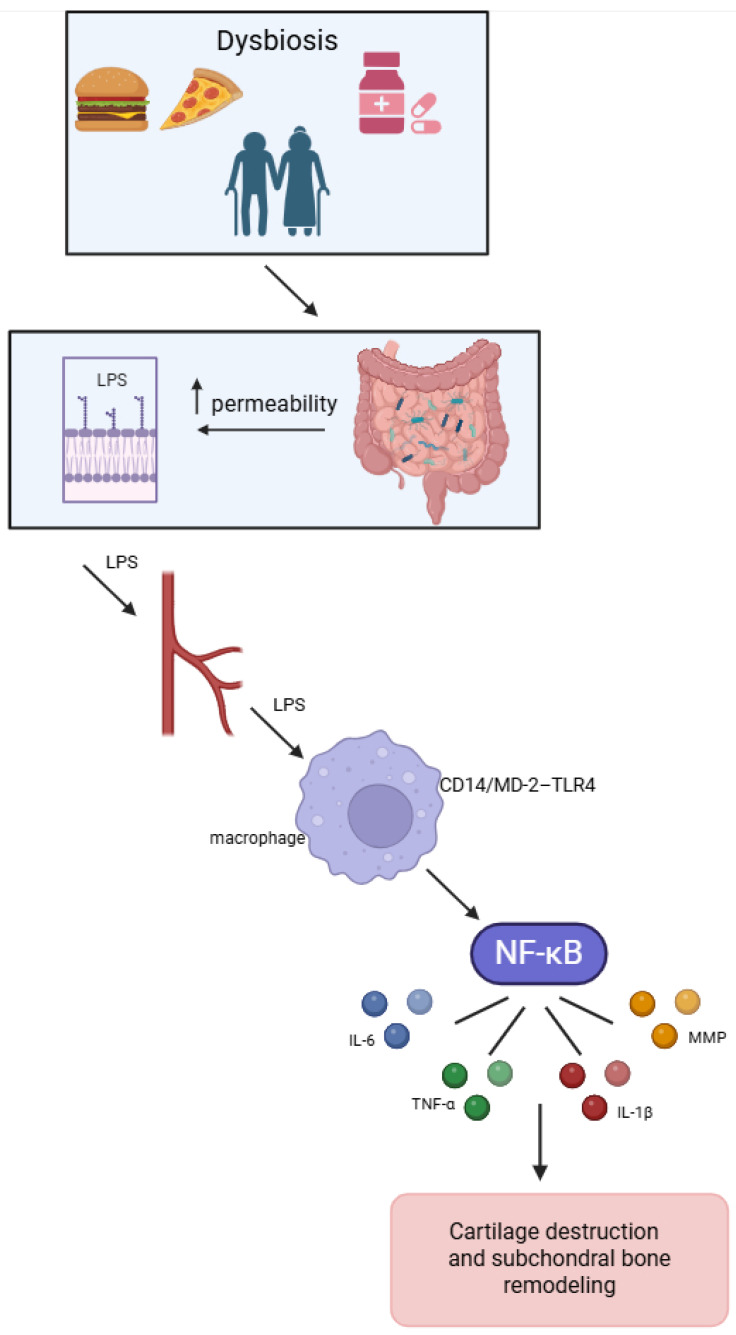
Influence of factors on the gut–joint axis. Dysbiosis associated with diet, ageing, obesity, or antibiotics disrupts tight junctions and increases intestinal permeability, allowing LPS to enter the circulation. Circulating LPS activates the CD14/MD-2-TLR4 complex on macrophages and chondrocytes, triggering the NFκB pathway and the production of pro-inflammatory cytokines (IL-1β, TNF-α, IL-6), and matrix-degrading enzymes (MMPs, ADAMTS). This process promotes cartilage damage and subchondral bone remodelling, linking gut dysbiosis to OA. Created in BioRender. Plewa, P. (2026) https://BioRender.com/gjz9ugc. LPS—lipopolysaccharide, IL—interleukin, TNF-α—tumour necrosis factor-α, NFκB—nuclear factor κB, MMP—matrix.

The gut–joint axis represents a mechanistic continuum through which the intestinal microbiota influence joint tissues via metabolic, immune, and endocrine pathways. Under physiological conditions, a balanced gut microbiome maintains epithelial integrity, produces anti-inflammatory metabolites, and promotes immune tolerance, collectively supporting systemic and articular homeostasis [17,18]. When dysbiosis arises as a result of diet, ageing, obesity, or antibiotic exposure, it disrupts intestinal tight junctions, increases permeability, and allows bacterial components such as lipopolysaccharide (LPS), a potent endotoxin from Gram-negative bacteria, to enter the systemic circulation [19]. Circulating LPS binds to the CD14/MD-2–Toll-like receptor 4 (TLR4) complex on synovial macrophages and chondrocytes, activating the nuclear factor κB (NFκB) pathway and inducing the production of pro-inflammatory cytokines (interleukin [IL]-1β, tumour necrosis factor-α [TNF-α], IL-6) and matrix-degrading enzymes (matrix metalloproteinases [MMPs] and a disintegrin and metalloproteinase with thrombospondin motifs [ADAMTS]). These processes promote cartilage destruction and subchondral bone remodelling [20,21]. This low-grade systemic inflammation forms the biological bridge between intestinal dysbiosis and joint degeneration (Figure 1).

**Figure 2:**
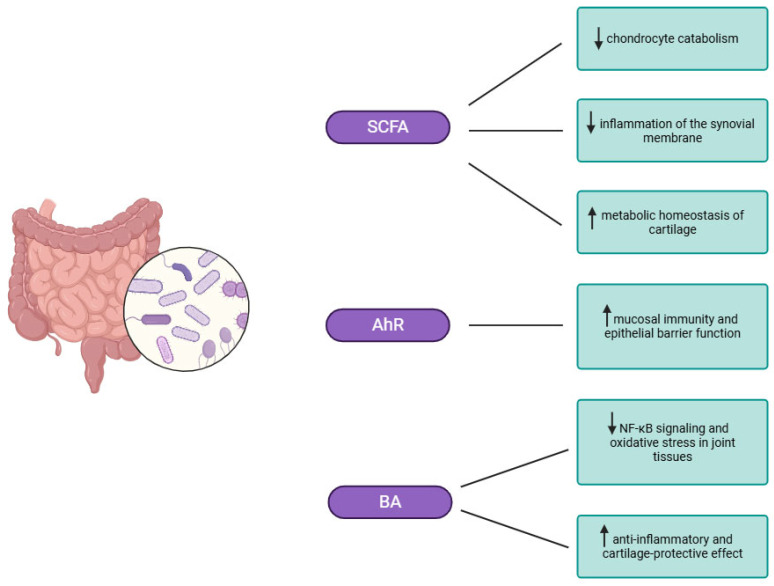
The influence of factors related to bacterial metabolism on the gut–joint axis. The anti-inflammatory gut microbiota produces SCFAs, which alleviate synovial inflammation, limit chondrocyte catabolism, and support cartilage metabolic homeostasis. Activation of AhR further regulates mucosal immunity and epithelial barrier function, supporting joint protection. Another mechanism linking the gut to joints is the modulation of BA, which exerts anti-inflammatory and cartilage-protective effects by inhibiting NF-κB and oxidative stress in joint tissues. Created in BioRender. Plewa, P. (2026) https://BioRender.com/bd6hzpe. SCFA—produces short-chain fatty acid, AhR—aryl hydrocarbon receptor, BA—bile acid.

Counteracting these pro-inflammatory signals, the gut microbiota produce short-chain fatty acids (SCFAs)—mainly butyrate, propionate, and acetate—through fermentation of dietary fibre. SCFAs reinforce intestinal tight junctions, limit bacterial translocation, and regulate immune balance via the G-protein-coupled receptors GPR41 and GPR43, enhancing regulatory T-cells (Treg) and suppressing T helper 17 (Th17) responses. Together, these effects reduce synovial inflammation, inhibit chondrocyte catabolism, and support cartilage metabolic homeostasis. Reduced SCFA production during dysbiosis removes this regulatory brake, amplifying the inflammatory cascade initiated by LPS–TLR4 activation (Figure 2). In addition, SCFA has an effect in other chronic joint diseases (dysbiosis in osteoporosis inhibits osteoclasts and increases bone mass, in the case of RA, disturbs the balance of immune cells) [22,23,24].

Another important pathway linking the gut and the joint involves microbial modulation of bile acid (BA) composition. Secondary BAs activate the farnesoid X receptor (FXR) and the G-protein-coupled BA receptor 1 (TGR5) in intestinal epithelial cells. FXR regulates lipid and glucose metabolism, while TGR5 activation stimulates secretion of glucagon-like peptide-1 (GLP-1) from enteroendocrine L-cells. GLP-1 exerts anti-inflammatory and chondroprotective effects, suppressing NF-κB signalling and oxidative stress in joint tissues. Restoration of the microbiota–BA–FXR/TGR5–GLP-1 pathway in experimental OA models has been shown to alleviate pain and cartilage degradation, confirming a functional gut–joint link (Figure 2) [25].

The aryl hydrocarbon receptor (AhR), a ligand-activated transcription factor sensing microbial metabolites, adds another regulatory layer. Tryptophan-derived indoles produced by commensal bacteria act as natural AhR ligands, modulating mucosal immunity and epithelial barrier function. In OA models, reduced microbial indole synthesis heightened chondrocyte inflammation, whereas supplementation with indole-3-propionic acid, an AhR agonist, inhibited NF-κB activity and attenuated disease progression [26,27]. Thus, SCFAs, secondary BAs, and indoles function as molecular messengers maintaining immunometabolic homeostasis; when their production declines, systemic inflammation extends to the joint microenvironment. Clinical and translational studies further support this concept—namely, that alterations in the gut microbiome can exert direct and measurable effects on joint inflammation, cartilage integrity, and pain in OA (Figure 2) [17,18,20].

In summary, microbial metabolites interact with host receptors, modulating key physiological and pathophysiological processes in both human health and disease. Over the past decade, numerous metabolite-receptor interactions mediated by gut microbiota have been identified. These systems play a crucial role in regulating immune responses and the development of inflammatory diseases. At the same time, a significant portion of microbiota-gut-host interactions remains unexplained. This is due to the enormous chemical diversity of microbial metabolites, thousands of which have not been functionally characterised [28].

### 3.2. Immune Response

The immune system serves as the key intermediary translating gut microbial imbalance into joint inflammation and tissue degeneration. Under physiological conditions, commensal microorganisms maintain intestinal homeostasis and peripheral immune tolerance by regulating the equilibrium between Th17 cells and Tregs and preventing pathological immune activation within the gut–joint axis [24,29]. Microbial metabolites such as SCFAs and tryptophan-derived indoles activate receptors, including G protein-coupled receptor 43 (GPR43) and AhR, promoting transcription of forkhead box P3 while suppressing retinoic acid receptor-related orphan receptor γt. When dysbiosis develops, the intestinal epithelial barrier becomes compromised, allowing microbial products such as LPS to enter the systemic circulation. LPS binding to TLR4 on macrophages, dendritic cells, synovial fibroblasts, and chondrocytes triggers the MyD88–NF-κB signalling cascade, increasing expression of cyclooxygenase-2, inducible nitric oxide synthase, and the pro-inflammatory cytokines IL-6 and TNF-α [30,31]. At the same time, microbial and danger-associated signals assemble the NOD-like receptor protein 3 (NLRP3) inflammasome, activating caspase-1 and converting pro–IL-1β and pro–IL-18 into their active forms. These cytokines amplify synovial inflammation, recruit immune cells, and intensify oxidative stress, accelerating articular cartilage degradation.

Within the synovial microenvironment, macrophage polarisation shifts towards the M1 phenotype, characterised by high production of reactive oxygen species, TNF-α, and IL-1β, while reparative M2 macrophages and Treg cells decline. The resulting pro-inflammatory milieu stimulates chondrocytes and synovial fibroblasts via NF-κB and mitogen-activated protein kinase signalling, upregulating MMPs (MMP-3, MMP-13) and ADAMTS that mediate cartilage matrix breakdown [21]. Adaptive immunity perpetuates this response: persistent cytokine signalling (IL-6, IL-1β) activates signal transducer and activator of transcription 3 (STAT3) in naïve T cells, promoting Th17 differentiation at the expense of Tregs. Increased IL-17A and IL-22 production subsequently induces synoviocytes to release further IL-6, granulocyte–macrophage colony-stimulating factor, and MMPs, reinforcing a chronic inflammatory circuit between gut and joint [24,29].

Systemically, repeated exposure to microbial ligands and cytokines reprogrammes monocytes and macrophages into a ‘trained immunity’ state characterised by enhanced glycolytic metabolism and epigenetic marks such as H3K4me3, which potentiate exaggerated inflammatory responses. This contributes to inflammaging—a persistent low-grade inflammatory phenotype associated with intestinal permeability and elevated C-reactive protein (CRP), IL-6, and TNF-α levels in patients with OA [32,33,34]. Such immune alterations maintain synovial activation and nociceptive sensitisation even in the absence of infection.

There is increasing discussion about how the gut microbiota may be connected with numerous diseases and disorders. It has been shown that a patient’s microbiome is strongly correlated with various health conditions [35]. One key question is whether, and in what way, the gut microbiota affects the pathophysiology of OA.

Many studies have demonstrated that the gut microbiota of patients with OA differs markedly from that of healthy individuals. These patients show alterations in both the composition and functionality of gut bacteria, as well as evidence of mild inflammation. Significant dysbiosis has been observed in OA. In healthy individuals, the gut microbiota consists not only of bacteria but also viruses, fungi, archaea, and protozoa [36]. Researchers have identified two broad groups of bacteria: those that appear protective against disease and those whose levels are elevated in patients with OA [37]. The first group includes the genera *Bacteroides*, *Agathobacter*, *Faecalibacterium*, and *Roseburia*. In particular, the family Methanobacteriaceae, the order Desulfo-vibrionales, and the genus subgroup *Ruminiclostridium* 5 have shown notable protective effects [38,39]. Similarly, *Bifidobacterium* is associated with a lower risk of OA. *Ruminococcaceae* UCG-003 and *Enterorhabdus* may reduce the risk of hip OA, while Methanobacteriaceae and Desulfovibrionales are negatively correlated with knee OA [39]. By contrast, patients with OA exhibit higher levels of *Acidaminococcus*, *Gordonibacter*, *Exiguobacterium*, *Lachnoclostridium*, Clostridiales, and Firmicutes [38,40]. *Streptococcus* and *Enterococcus* have been linked to increased pain in patients with OA, possibly due to microcellular vesicles produced by *Streptococcus* spp. in the gastrointestinal tract [41,42]. Supporting the relevance of microbiota composition, supplementation with probiotics has been reported to help patients with OA manage pain and inflammation [41,42].

The Gram-negative portion of the gut microflora is a major reservoir of LPS. Physiologically, LPS from the intestines is transported into the bloodstream, activating the immune system and causing low-grade inflammation [43,44]. It has been suggested that LPS may contribute to OA development through pro-inflammatory innate immune responses, particularly in combination with joint injuries. Reducing LPS levels in the body may therefore help prevent or treat OA [45].

SCFAs are the main products of gut microbial fermentation. Microbiome disturbances affect both the types and quantities of these metabolic products. Certain SCFAs—acetic acid, propionic acid, and butyric acid—have been shown to play roles in bone metabolism [46]. Reduced SCFA synthesis results in increased inflammatory factors in the blood and synovial fluid [47]. Gut microflora also plays an important role in the production of 5-hydroxytryptamine (5-HT); gut bacteria can stimulate chromaffin cells to release 5-HT [48]. Because 5-HT affects bone metabolism, disturbances in intestinal microflora alter 5-HT levels and disrupt the balance between bone resorption and formation [49]. Other metabolites differing between patients with OA and healthy controls include pyrogallol, which impairs staphylococcal biofilm formation by inducing bacterial oxidative stress, and 3-hydroxybutyrate, which has been shown to ameliorate OA through activation of the ERBB3 signalling pathway in preclinical studies and by reducing cartilage degeneration via the HCAR2/AMPK/PINK1/Parkin pathway [50,51,52]. Levels of these metabolites are lower in patients with OA. Likewise, decreases in docosapentaenoic acid, naringenin, and pregnenolone are associated with OA progression [38].

For many years, synovial fluid was believed to be a sterile environment. However, recent studies have reported the presence of microorganisms in synovial fluid, challenging this assumption. These microorganisms may be involved in the pathogenesis and progression of OA [53]. Supporting the ‘leaky gut’ hypothesis, some bacteria found in the intestines, such as *Enterococcus faecium* and *Staphylococcus hominis,* have also been detected in synovial fluid [54,55,56]. This observation may further suggest that probiotic supplementation could help relieve joint pain [57]. It should be noted that synovial fluid from OA patients primarily contains bacterial DNA and cell wall fragments, such as peptidoglycan, which can stimulate the immune system and induce inflammation. Current molecular methods, such as 16S rRNA sequencing, do not allow for the unequivocal determination of bacteria as being alive and biologically active. Isolation of live bacteria from synovial fluid is very rare, so most evidence points to the role of microbial fragments and their metabolites, not colonisation with live bacteria. Confirmation of activity would require culture, RNA assays, or methods that distinguish between live and dead cells [58]

## 4. Research Review: Animal and Human Research

### 4.1. The Gut–Joint Axis in OA

A growing body of research has established a link between OA and a gut–joint axis, indicating that intestinal dysbiosis, barrier dysfunction, and low-grade systemic inflammation can contribute to the symptoms and structural changes associated with OA [17].

A substantial number of narrative and systematic reviews have converged on a stereotyped dysbiosis, characterised by a depletion of SCFA producers and an enrichment of pro-inflammatory or pathobiont taxa [37]. The plausibility of this hypothesis is supported by several pathways, including LPS translocation, macrophage and T-cell activation, and cytokine signalling [37].

Two independent human cohorts with symptomatic hand OA demonstrated distinct microbiome-related metabolomic signatures of gut involvement in OA. In the community-based discovery cohort (XO Study) (*n* = 1359, among whom 70 had symptomatic hand OA), shotgun metagenomics revealed a depletion of gut microbial tryptophan-biosynthesis functions accompanied by taxonomic shifts. These included higher levels of *Bilophila wadsworthia*, *Hungatella hathewayi*, *Lactobacillus mucosae*, and *Citrobacter koseri,* and reduced *Roseburia intestinalis*, *Bacteroides* spp., and *Haemophilus* spp., compared with controls. These functional losses co-occurred with a distinct plasma signature characterised by elevated serotonin-pathway products (e.g., 5-HIAA, 5-HTOL) and diminished indole/kynurenine-pathway metabolites (e.g., indole-3-lactic acid [ILA], skatole, 3-hydroxyanthranilic acid), supporting a microbiome–tryptophan axis hypothesis [59]. In the independent, urban-matched case–control cohort (71 with symptomatic hand OA vs. 71 controls), the key replicated feature was persistently lower circulating ILA. Multi-omics from the discovery cohort linked lower ILA to reduced microbial tryptophan-biosynthesis capacity and to diminished *Bacteroides mediterraneensis* [59].

Mechanistically, transplantation of faecal microbiota from donors with impaired metabolic function has been shown to accelerate OA progression in murine models, providing causal support for the proposed axis [60].

Recent studies further highlight consistency in clinical correlates. Notably, higher levels of gut *Streptococcus* have been associated with increased pain on the Western Ontario and McMaster Universities Arthritis Index (WOMAC) pain subscale—a validated patient-reported outcome measure for hip and knee OA. In the population-based Rotterdam Study III (*n* = 1444 participants with hip and/or knee OA), gut profiling linked a greater relative abundance of *Streptococcus* species to higher WOMAC pain scores. A plausible mechanistic pathway is leaky-gut endotoxaemia: increased intestinal permeability allows gut-derived products, particularly LPS, to prime synovial macrophage-driven inflammation. Circulating LPS burden correlates with OA severity and with higher WOMAC scores. Taken together, as outlined in this review, these findings support a clinically meaningful microbiome–symptom axis in OA, although the evidence remains observational and therefore hypothesis-generating. These associations are susceptible to confounding by adiposity, diet, and medication use (e.g., non-steroidal anti-inflammatory drugs) and should be analysed with careful adjustment in observational designs [61].

Further work indicates that persistent knee pain following total knee replacement is associated with distinct gut microbial patterns, suggesting potential links between specific pain phenotypes and dysbiosis [62].

Across human OA cohorts, both the gut and joint-adjacent microbiomes display recurrent patterns marked by expansion of Gram-negative Proteobacteria, including Enterobacteriaceae such as *Escherichia*, *Klebsiella*, and *Shigella*. In several datasets, higher levels of Actinobacteria are also observed, alongside a contraction of SCFA-producing commensals (e.g., *Roseburia*, *Faecalibacterium*, Ruminococcaceae, *Subdoligranulum*, *Agathobacter*) [63].

Reviews consistently report decreased abundance of SCFA-producing genera such as *Faecalibacterium*, *Roseburia*, and *Butyricicoccus*, taxa linked to epithelial integrity and an anti-inflammatory milieu [37].

Although intervention data remain preliminary, evidence is gradually accumulating. A 2024 meta-analysis of clinical trials reported that oral probiotics provided moderate improvements in pain and inflammation, while emphasising heterogeneity in strains and study protocols [64].

A randomised controlled trial published in the *British Journal of Nutrition* (2024) found that a multistrain probiotic reduced disease severity and improved postural balance, with preliminary indications of enhanced intestinal barrier function [65].

A *Scientific Reports* randomised controlled trial testing *Latilactobacillus sakei* LB-P12 demonstrated WOMAC improvement over placebo across 12 weeks (exploratory design with good safety) [66].

Likewise, a 2025 meta-analysis focused on knee OA suggested that probiotics may improve pain, function, and inflammatory markers, while calling for larger, standardised trials [67].

Contemporary overviews highlight the clinical plausibility of the model but also delineate key evidence gaps, including the need to understand strain-specific effects, to identify likely responders (e.g., metabolic or inflammatory phenotypes), and to standardise endpoints such as permeability and metabolomic outputs [63]. Emerging mechanistic work, particularly involving BA/FXR–GLP-1 signalling through the gut, further supports therapeutic targeting of the axis. Across two human cohorts, reduced glycoursodeoxycholic acid was identified as a bile-acid signature of OA. In mice, intestine-restricted FXR suppression enhanced enteroendocrine GLP-1 production and mitigated cartilage damage; these effects were abolished by GLP-1R blockade and mimicked by GLP-1R agonism. Reshaping the BA pool using FDA-approved ursodeoxycholic acid (UDCA) or colonisation with *Clostridium bolteae*, which promoted UDCA/GUDCA formation, alleviated OA in mice. UDCA use was also associated with a lower risk of OA-related joint replacement in humans. However, these findings remain limited by their preclinical origins and by the observational nature of the human data. Causality in people remains unproven, residual confounding cannot be excluded, and generalisability beyond the studied cohorts will require randomised, mechanistically anchored trials [25]. The pro-eubiosis vs. dysbiosis profile in osteoarthritis is presented in Table 1.

### 4.2. Animal Studies: OA Models and Gut Microbiota Transplantation

Across a range of murine models, direct manipulations of the gut microbiota, including germ-free rearing and faecal microbiota transplantation have been shown to causally modulate the severity of OA [60,68]. In the destabilisation of the medial meniscus mouse model, germ-free mice exhibit markedly reduced cartilage degeneration and proteoglycan loss compared with specific-pathogen-free controls. They also form smaller osteophytes and show lower plasma LPS-binding protein levels. These findings suggest that exposure to microbiota-derived products plays a pivotal role in the development of structural joint damage [68]. Translational faecal microbiota transplantation experiments provide further evidence supporting this hypothesis: the transfer of stool from donors with knee OA and metabolic syndrome into germ-free mice prior to meniscal or ligament injury leads to augmented histological OA severity and synovitis compared with transplants from healthy donors or OA donors without metabolic syndrome [60]. Recipients of the metabolic-syndrome OA microbiome demonstrate heightened gut permeability, reduced tight-junction transcripts [69], and elevated circulating LPS consistent with a barrier-to-inflammation mechanism that amplifies joint damage [60]. Furthermore, host susceptibility to OA appears to be microbiome-mediated. Strain-dependent differences in OA risk are transferable, alongside immunophenotypic shifts [70]. Conversely, broad-spectrum antibiotic depletion of intestinal bacteria attenuates OA induced by destabilisation of the medial meniscus (DMM) and lowers systemic LPS and inflammatory readouts in mice [71]. Finally, the potential protective role of repopulation strategies is highlighted by probiotic intervention following depletion, which has been shown to reduce DMM-induced cartilage damage and slow disease progression, further supporting a causal gut–joint axis [72].

### 4.3. Diet Microbiome Interventions Modulate OA Progression

Diet functions as one of the most potent and modifiable regulators of the gut microbiome, intestinal barrier integrity, and systemic inflammation, forming a mechanistic bridge between metabolism and joint degeneration through the gut–joint axis.

Among dietary models, the Mediterranean diet (MedDiet)—rich in fibre, polyphenols, legumes, fruits, vegetables, and unsaturated fatty acids—consistently demonstrates beneficial associations with OA. In a recent meta-analysis, Veronese et al. (2024) confirmed that higher adherence to the MedDiet was associated with a significantly lower risk of symptomatic knee OA and improved WOMAC pain and function scores [73]. A magnetic resonance imaging–based cohort study from the Osteoarthritis Initiative reported that individuals with sustained high MedDiet adherence over 8 years experienced slower cartilage loss and lower synovial inflammation, suggesting potential structure-modifying properties [74]. Mechanistically, MedDiet patterns promote the abundance of *Faecalibacterium* and *Roseburia*—saccharolytic genera producing SCFAs such as butyrate and propionate—which reinforce tight-junction proteins, suppress LPS translocation, and attenuate low-grade inflammation via GPR41/43-dependent signalling [23,75].

By contrast, Western-style diets high in saturated fats and refined carbohydrates but low in fibre induce dysbiosis dominated by LPS-producing Gram-negative bacteria, thinning of the mucus layer, and increased intestinal permeability. In high-fat-fed mice, dysbiosis accelerated cartilage degradation and subchondral bone remodelling relative to chow-fed controls [76]. Translational evidence parallels these findings: in a 2025 randomised crossover study of 40 older adults with obesity, just 2 weeks of a high-fat diet raised postprandial LPS and zonulin concentrations by approximately 35%, confirming transient barrier dysfunction and diet-induced metabolic endotoxaemia [77]. Evidence for microbiome-targeted dietary strategies is strengthening. In a 6-month randomised controlled trial, Fortuna et al. (2024) reported that daily supplementation with oligofructose-enriched inulin in 120 overweight adults with knee OA improved objective physical function (Timed Up-and-Go and 40 m walk tests), reduced trunk fat, and increased faecal abundance of *Faecalibacterium prausnitzii* and butyrate levels, confirming a causal fibre–microbiome–metabolic link [78]. Lei et al. (2017) demonstrated that *Lactobacillus casei* Shirota supplementation reduced knee OA pain and stiffness compared with placebo in a double-blind randomised controlled trial of 180 adults [76]. More recently, Karim et al. (2024) reported that a multi-strain probiotic restored intestinal barrier markers and reduced serum zonulin in patients with OA, aligning with improved pain and function scores [65]. The degree of food processing also shapes microbiome–host interactions. In a large UK Biobank cohort (>160,000 participants), Wei et al. (2024) observed that every 10% increase in ultra-processed food intake corresponded to a 10% higher risk of symptomatic knee OA, independent of body mass index and age [79]. Magnetic resonance imaging analyses from the same cohort linked ultra-processed food intake to greater cartilage thinning and subchondral bone lesions [80]. Daniel et al. (2024) later confirmed that inflammatory responses to carboxymethylcellulose vary by baseline microbiome composition, revealing personalised susceptibility to dietary emulsifiers [81].

The influence of fatty acid quality has also received attention. A meta-analysis of nine randomised trials by Deng et al. (2023) found modest but statistically significant pain relief with omega-3 polyunsaturated fatty acid supplementation [82].

Weight-reducing interventions integrate microbial, metabolic, and mechanical pathways. In a 12-week pilot trial, a fibre-enriched hypocaloric diet lowered CRP, improved stool SCFA-to-branched-chain amino acid ratios, and alleviated knee pain in overweight patients with OA [75]. In the STEP-9 randomised trial published in *The New England Journal of Medicine*, Bliddal et al. (2024) showed that once-weekly semaglutide 2.4 mg resulted in a mean 13.7% weight reduction and significantly improved WOMAC pain scores in obese individuals with knee OA [83]. Beyond mechanical unloading, GLP-1 receptor activation enhanced intestinal motility, strengthened epithelial junctions, and down-regulated macrophage cytokine release, positioning the intestine as an active metabolic–inflammatory interface influencing joint homeostasis.

Collectively, these findings establish diet as a central determinant of OA pathogenesis through microbiome-dependent mechanisms. High-fibre and Mediterranean-style dietary patterns promote microbial diversity and SCFA signalling; limiting saturated fats and ultra-processed foods prevents endotoxaemia; and sustainable weight loss, whether behavioural or pharmacological, reduces both metabolic and inflammatory stress on the joint. Prebiotic, probiotic, and synbiotic interventions already demonstrate functional improvements, although larger and longer trials are required to determine their structural joint benefits [23,65,74,75,76,77,78,79,80,81,82,83,84,85,86].

### 4.4. Imaging Correlates of the Gut–Joint Axis in OA

This section synthesises current evidence on radiologic correlates of the gut–joint axis in knee OA, examining whether imaging markers of synovial inflammation and cartilage integrity vary with gut and circulating microbial profiles. The aim is to delineate clinically relevant imaging differences that mirror microbiome patterns.

In a cross-sectional cohort of 40 patients with primary knee OA, ultrasound-measured femoral cartilage thickness (FCT) demonstrated anatomy-specific associations with microbial signatures detected via quantitative polymerase chain reaction in blood and synovial fluid. Lateral FCT was inversely associated with the relative abundance of Firmicutes in both compartments. Moreover, higher circulating levels of Proteobacteria and Actinobacteria correlated with thinner lateral cartilage. No significant associations were observed between medial or intercondylar FCT and any of the tested phyla. Serum LPS concentrations exceeded those in synovial fluid, yet neither compartment’s LPS levels correlated with FCT, suggesting that the imaging signal relates more to phylum composition than to endotoxin burden [87].

Ultrasound-defined knee synovitis—operationalised as synovial hypertrophy ≥ 4 mm and/or a positive power Doppler (PD) signal is also associated with specific gut microbial features. In a large community cohort, participants with synovitis displayed a distinct gut fungal composition compared with those without synovitis, with a stepwise gradient across imaging categories (control → non-PD synovitis → PD-positive synovitis). *Schizophyllum* abundance exhibited inverse associations with both the presence and activity of ultrasound-detected synovitis, with lower levels corresponding to more active (PD-positive) inflammation. By contrast, global bacterial diversity metrics (α and β diversity) did not differ by ultrasound category, although selected bacterial taxa showed directionally consistent associations with synovitis in adjusted analyses. Collectively, these findings suggest a link between the gut mycobiome and its interaction with the bacteriome and the ultrasonographic phenotype of knee synovitis. Because the data are cross-sectional and associative, they should be interpreted as supporting a gut–joint connection rather than demonstrating causality [88].

Given that both datasets are cross-sectional and subject to confounding, they indicate that microbiome signals are reflected in imaging phenotypes, yet do not establish temporal or causal relationships. Accordingly, prospective, standardised studies are required.

## 5. Conclusions

A growing body of research indicates that osteoarthritis (OA) is not solely a consequence of mechanical wear and tear but is also influenced by disturbances in the gut–joint axis, including gut dysbiosis, increased intestinal permeability, and inflammation. A characteristic microbiome pattern is observed in many human cohorts: a reduction in the number of bacteria producing short-chain fatty acids (SCFAs) and a concomitant increase in pro-inflammatory taxa, which correlates with pain severity (e.g., WOMAC score) and structural changes in joints. Studies have highlighted the important role of tryptophan and bile acid metabolism, suggesting potential mechanistic pathways linking the microbiome to degenerative processes in joints. Animal models provide evidence that the microbiome can modulate both the development and severity of osteoarthritis. Microbiota transplantation from affected donors exacerbates joint lesions, while probiotic and prebiotic interventions, dietary modification, and bile acid modulation reduce cartilage damage. Imaging studies also suggest that microbiome profiles are reflected in synovial phenotypes and cartilage thickness.

Despite promising clinical and experimental data, most human studies are observational. The clinical application of microbiome-targeted interventions in osteoarthritis faces significant challenges. Therapeutic effects exhibit significant heterogeneity, depending on the strain or combination of strains used, the dose, duration of intervention, and the initial composition of the host microbiome. Growing evidence indicates that the response to probiotics, prebiotics, and dietary modifications is highly individualised and modulated by factors such as metabolic phenotype, obesity status, inflammatory status, medication use, and genetic predisposition. It is also important to emphasise that a significant portion of the mechanistic evidence supporting the role of the gut–joint axis and the effectiveness of specific microbiome interventions comes from preclinical studies, primarily animal models, whose direct translation to the human population remains limited. Furthermore, most available clinical trials are characterised by short follow-up periods, preventing a clear assessment of long-term safety and the durability of clinical and structural effects. The lack of standardised protocols, endpoints, and biomarkers of response further complicates the comparison of results between studies.

Therefore, future research should focus on three priority areas:

1. Mechanistic studies—precisely determining how the microbiome and its metabolites influence inflammatory and degenerative processes in joints.
2. Clinical trial design—standardisation of protocols, long-term follow-up, and identification of safe and effective endpoints and biomarkers of response.
3. Individualised intervention strategies—identifying predictors of patient response to probiotics, prebiotics, dietary modulation, and other microbiome approaches, taking into account metabolic phenotype, inflammatory status, medications, and genetic factors.

This approach will both confirm the causal role of the microbiome in osteoarthritis and enable the safe and effective implementation of microbiome strategies in clinical practice. This review also highlights the importance of understanding the interactions between the immune system, microorganisms, and their metabolites, as these processes can determine joint health and resilience.

## Figures and Tables

**Table 1:** Pro-eubiosis versus dysbiosis profiles in OA.

Category	Representative Taxa	Typical Signal in OA Literature	Sources
Protective, barrier-supporting taxa			
	*Faecalibacterium* (e.g., *F. prausnitzii*)	Butyrate-producing taxon	[37]
		Anti-inflammatory, epithelial support	
		Often reduced in OA	
	*Roseburia*, *Butyricicoccus*	Butyrate-producing taxon	[37]
		Often reduced in OA	
	*Bifidobacterium*	Pro-eubiosis signal	[64]
		Frequently included in OA probiotic formulations	
	*Akkermansia*	Supports barrier/mucus interface and metabolic homeostasis	[37]
		Discussed as potentially protective in OA-focused reviews	
	Ruminococcaceae	Butyrate-producing taxon	[63]
		Often reduced in OA	
	*Subdoligranulum*	Butyrate-producing taxon	[63]
		Often reduced in OA	
	*Agathobacter*	Butyrate-producing taxon	[63]
		Often reduced in OA	
	*Bacteroides* spp.	Often reduced in OA	[59]
	*Haemophilus* spp.	Often reduced in OA	[59]
Dysbiosis-associated, endotoxin-rich taxa			
	*Streptococcus* spp.	Positive correlation with WOMAC pain	[61]
	Enterobacteriaceae	LPS-rich pathobionts	[63]
		Over-represented in OA cohorts	
	Proteobacteria	Broad dysbiosis marker	[63]
		Frequently increased in OA	
	*Collinsella and Prevotella/Ruminococcus*	Reported associations with pro-inflammatory/metabolic profiles in subsets	[37]
		Findings vary by study	
	*Pseudomonas*	Frequently increased in OA	[63]
	*Bilophila wadsworthia*	Frequently increased in OA	[59]
	*Hungatella hathewayi*	Frequently increased in OA	[59]
	*Lactobacillus mucosae*	Frequently increased in OA	[59]
	*Citrobacter koseri*	Frequently increased in OA	[59]

## Data Availability

No new data were created or analysed in this study.

## Abbreviations

The following abbreviations are used in this manuscript:

ADAMTS	A disintegrin and metalloproteinase with thrombospondin motifs
AhR	Aryl hydrocarbon receptor
BA	Bile acid
CRP	C-reactive protein
DMM	Destabilisation of the medial meniscus
IL	Interleukin
FCT	Femoral cartilage thickness
FXR	Farnesoid X receptor
GLP-1	Glucagon-like peptide-1
GPR43	G protein-coupled receptor 43
LPS	Lipopolysaccharide
MMP	Matrix metalloproteinases
NFκB	Nuclear factor κB
NLRP3	NOD-like receptor protein 3
OA	Osteoarthritis
SCFA	Short-chain fatty acids
STAT	Signal transducer and activator of transcription
Th	T helper
TLR	Toll-like receptor
Treg	Regulatory T-cells
UDCA	Ursodeoxycholic acid
